# Whole body MRI by MY-RADS for imaging response assessment in multiple myeloma

**DOI:** 10.1038/s41408-025-01327-4

**Published:** 2025-07-17

**Authors:** Christina Messiou, Nuria Porta, Dow-Mu Koh, Angela Riddell, Katherine Downey, James Croft, Leonora Conneely, Georgina Hopkinson, Alina Dragan, Tommy Brown, Simon Stern, Betty Cheung, Charalampia Kyriakou, Pawel Kaczmarek, Kevin Boyd, Charlotte Pawlyn, Jessica Winfield, Martin F. Kaiser

**Affiliations:** 1 The Royal Marsden Hospital Foundation NHS Trust, London, UK; 2 The Institute of Cancer Research, London, UK; 3 Epsom and St Helier University Hospitals, London, UK; 4 Croydon University Hospital, London, UK; 5 University College London Hospitals NHS Foundation Trust, London, UK; 6 Surrey and Sussex Healthcare NHS Trust, Brighton, UK

**Keywords:** Myeloma, Cancer imaging

## Abstract

Minimal residual disease (MRD) testing has underpinned the evaluation and expansion of therapeutic options for patients with multiple myeloma (MM). Imaging is essential for evaluating residual disease status, overcoming sampling errors inherent with other MRD modalities. The accuracy of whole-body MRI (WB-MRI) has led to its incorporation into MM diagnostic imaging guidelines. We report here on the prospective iTIMM trial (image-guided theranostics in MM; NCT02403102), designed to evaluate imaging residual disease using contemporary, functional WB-MRI as per MY-RADS protocol. In iTIMM, 70 MM patients planned to undergo autologous stem cell transplantation ASCT in newly diagnosed MM or at first relapse, underwent WB-MRI before start of induction and at day 100 post-ASCT. Patients with residual disease post-ASCT (RAC2 or higher) had shorter progression-free survival (median 24 months, 95% confidence interval (CI): 19–41 vs. 42 months, 95% CI: 37–not evaluable (NE), log-rank *p* = 0.013; hazard ratio (HR) 2.09 (95% CI: 1.15–3.78) and overall survival (median 47 months, 95% CI: 30.9–NE vs. NE (95% CI: NE–NE), *p* = 0.002, HR = 5.45 (95% CI: 1.67–17.87) than those without (RAC1). Imaging response also refined the prognostic association of bone marrow MRD and serological response. Our results support WB-MRI implementation for evaluation of residual disease alongside conventional laboratory-based assessments.

## Highlights

- WB-MRI imaging response as per MY-RADS criteria provides independent information to matched serological response and bone marrow minimal residual disease assessments.
- WB-MRI residual disease detection post-treatment as per MY-RADS criteria is associated with shorter PFS and shorter OS in this prospective trial.

## Introduction

The introduction of consensus criteria for minimal residual disease (MRD) testing for patients with multiple myeloma (MM) by the International Myeloma Working Group (IMWG) in 2016 has been a critical enabler towards better understanding of the relationship between depth of response and outcome [1]. MRD testing through cell or molecular-based assays has provided much more granular interrogation of depth of response than conventional complete response criteria and has underpinned the evaluation and expansion of therapeutic options now available to patients with MM.

Heterogeneity of plasma cell infiltration in the marrow space leading to sampling error is now well recognised [2] and therefore the IMWG recommends the use of imaging as an orthogonal evaluation of residual disease [1]. Furthermore, laboratory-based testing for MRD is not able to detect extramedullary disease (EMD), which has an incidence between 0.5–4.8% at new diagnosis, while in relapsed/refractory disease, the reported incidence is higher at 3.4–14% [3]. To date, much of the evidence supporting the use of imaging has centred on FDG PET/CT [4]. The inferior outcome for patients with positive scans, even in those achieving laboratory-based responses, reinforces the role for imaging [5, 6]. The high sensitivity of whole-body magnetic resonance imaging (WB-MRI) is now well recognised and reflected in imaging guidelines for myeloma diagnosis and surveillance of patients with asymptomatic myeloma [7–9]. Historical arguments that MRI-based response assessments lag behind FDG PET/CT and lack specificity are no longer relevant in an era of contemporary MRI. In 2019, the MY-RADS consensus established recommendations for acquisition and reporting of WB-MRI for myeloma and included quantitative/functional diffusion-weighted and Dixon MRI techniques as standard [10]. Furthermore, the implementation of highly sensitive WB-MRI for myeloma diagnosis is resulting in increased use throughout the patient pathway [11–13].

To address uncertainties and define the role of WB-MRI in myeloma, we conducted, to our knowledge, the first prospective study to evaluate the detection of residual disease using contemporary WB-MRI protocols and the correlation with serum and marrow estimates of disease and patient outcome.

## Materials and methods

### Trial design

We conducted a single-centre observational study of baseline WB-MRI and FDG PET/CT in prospectively recruited patients with either a new diagnosis of symptomatic MM or at first relapse, who were planned to undergo induction therapy and high-dose melphalan and autologous stem cell transplant (ASCT), with WB-MRI follow-up at 3 months (day 100) post autograft and 2-year follow-up for progression-free survival (iTIMM, Image-guided theranostics in MM, NCT02403102). Institutional review board and national ethics committee approval were obtained. Written informed consent was obtained from all patients. Patients were recruited between 11 November 2015 and 03 March 2018. Baseline comparison and superior sensitivity of WB-MRI in iTIMM have previously been reported [7]. We report here on the correlation between WB-MRI response findings at 3 months post-ASCT and outcomes.

### Patient population

Inclusion criteria were patients over the age of 18 with a new diagnosis of MM or disease at first relapse, planned for high-dose melphalan and ASCT. Diagnosis and relapse were defined as per IMWG Criteria using laboratory parameters, including bone marrow trephine and assessment of end-organ damage [1, 14, 15].

Patients with a diagnosis of another malignancy in the prior 5 years or contraindications to MRI or FDG PET/CT were excluded. Cytogenetic analysis was performed by the Royal Marsden Hospital’s Haematologic Malignancies Diagnostic Service (HMDS), including testing for t(4;14), t(14;16), t(14;20), del(1p), gain(1q) and del(17p) for all patients. Day 100 post-ASCT bone marrow biopsy and MRD assessment were performed by HMDS by flow cytometry with a sensitivity of 10^−4^, using a six-colour panel, as previously reported [16].

### MRI protocol

WB-MRI was performed using a Siemens Avanto 1.5 T system in line with the MY-RADS consensus document [10]. T_1_w and T_2_w sagittal spine images were acquired, followed by axial diffusion-weighted sequences covering head to knees and axial 3D gradient-echo Dixon sequences, as previously published [7]. The average total acquisition time was 45 minutes.

### Image analysis

WB-MRI scans were scored by 2 blinded, experienced reporting radiologists (>5 years’ experience each) and a final score was achieved by consensus. Discrepant cases were brought back to the readers for consensus by a third independent reader. Focal and diffuse disease on MRI were defined as per the MY-RADS criteria and scored 1–5 for response using the Response Assessment Criteria (RAC) where RAC1 = highly likely to be responding; RAC2 = likely to be responding; RAC3 = stable; RAC4 = likely to be progressing and RAC5 = highly likely to be progressing [10]. Focal disease and diffuse disease can coexist and are not mutually exclusive.

### Statistical methods

We explored the predictive value of image response by WB-MRI as per MY-RADS criteria at day 100 post-ASCT on outcome in terms of progression-free survival (PFS) and overall survival (OS) with the data collected in this prospective study. PFS was defined as time from stem cell re-infusion to progression as per IMWG criteria or death by any cause, and OS as time from stem cell re-infusion to death by any cause. Kaplan–Meier curves were generated, and disease burden groups were compared using the log-rank test. Associations were quantified on univariate and multivariate analyses of prognostic imaging and clinical factors at baseline and 3 months post-autograft. Cox-proportional hazard regression was used to estimate univariate and multivariate hazard ratios (HR) and 95% confidence intervals (CI). *P*-values < 0.05 were considered statistically significant. Associations between different response criteria at 3 months post-autograft were assessed by Pearson’s Chi-squared. Statistical analysis was performed in R (version 4.4.2), including packages survminer and gtsummary. The exploratory endpoint of iTIMM, comparing the burden of disease by WB-MRI and matched FDG PET/CT has been previously published [7].

The study was approved by the National Health Service (NHS) National Research Ethics Committee London–Surrey (NREC; 15/LO/0036) and conducted in accordance with the Declaration of Helsinki.

## Results

### Patient population

Between May 2015 and June 2018, 105 MM patients fit for and intended to undergo standard of care (SoC) high-dose melphalan chemotherapy and ASCT were recruited to the study. Patients were eligible to join the study either at the time of diagnosis of symptomatic MM or at the time of first relapse, if a second-line ASCT was planned. Inclusion was permissive for a real-world population; exclusion criteria were claustrophobia, implants where MRI was contraindicated and diagnosis of another malignancy within the last 5 years.

**Fig. 1:**
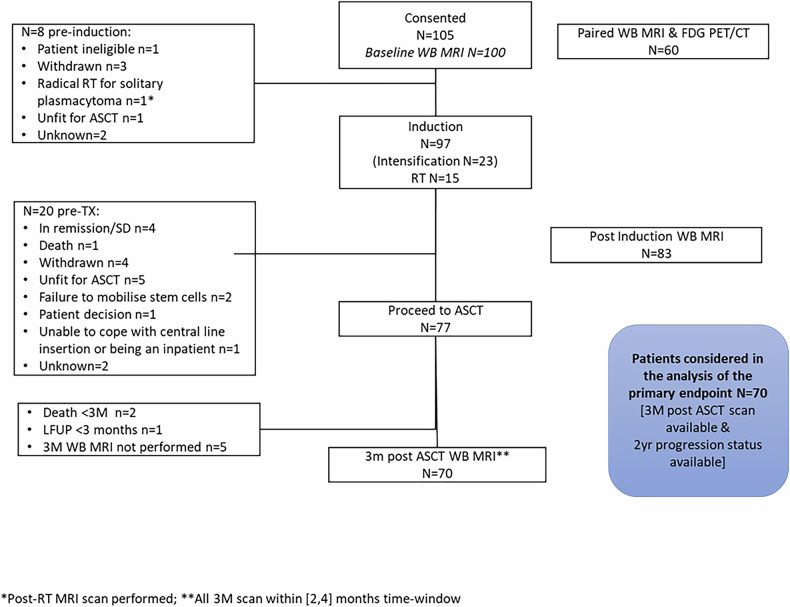
CONSORT diagram of the iTIMM trial. The patient flow from patients considered and screened at trial entry to the patient population eligible for primary endpoint analysis is depicted from top to bottom.

Patients were evaluable for the primary endpoint as per the pre-defined statistical analysis plan, if they had a day 100 post-ASCT WB-MRI scan and had been followed up for at least 2 years for progression (not lost to follow-up before). Of the 105 patients, 70 (66.7%) fulfilled these criteria and were included in the analysis. There were a range of reasons for ineligibility for analysis, with withdrawal from study (*n* = 7), patient unfit for ASCT (*n* = 6) and post-ASCT WB-MRI not performed (*n* = 5) being the most common reasons, reflecting the permissive inclusion criteria and real-world patient population (Fig. 1). Of note, indication for SoC ASCT in the UK NHS was based on physician assessment of patient fitness, not numerical age, with no formal upper age limit for the procedure. Patients included in the primary analysis group were comparable to the overall study population regarding clinical baseline parameters, apart from kidney function: 8/35 (22.9%) non-evaluable vs 5/70 (7.1%) evaluable patients had raised creatinine at study entry.

Patients in the primary analysis cohort (*n* = 70; Table 1) were predominantly male (61.4%), with a median age of 62 years (interquartile range (IQR) 57–67). The majority of patients (*n* = 54) had newly diagnosed myeloma (NDMM), whereas 16 patients were included at first relapse. For patients included at first relapse, the median time from diagnosis to first relapse was 53 months (IQR 25–57). Median follow-up after ASCT was 42 months (IQR 34–49). For 55/70 patients, a full complement of high-risk cytogenetic abnormality (HRCA) results for t(4;14), t(14;16), t(14;20), del(1p), gain(1q), del(17p) was available. Of these, 31 (56%) had no genetic abnormality, 17 (31%) had 1 HRCA (single hit), and 7 (13%) 2 + HRCA (double hit).

**Table 1:** Baseline and treatment characteristics.

Characteristic	*N* = 70^1^
Clinical characteristics	
Sex	
Female	27 (39%)
Male	43 (61%)
Age (years; median (IQR))	62 (57, 67)
Laboratory/staging characteristics	
Paraprotein type	
IgG	58 (82.9%)
IgA	6 (8.6%)
Light chain only	6 (8.6%)
Light chain type	
kappa	27 (38.6%)
lambda	43 (61.4%)
ISS stage	
1	33 (52%)
2	26 (41%)
3	5 (7.8%)
Unknown	6
Bone marrow trephine overall cellularity description by pathologist	
Normocellular	33 (52%)
Hypercellular	26 (41%)
Hypocellular	5 (7.8%)
No trephine performed	6
Plasma cells % of nucleated cells on trephine (median (IQR))	50.0 (30, 70)
Genetic profiling results	
t (4;14)	5 (9.1%)
t (14;16)	3 (5.5%)
t (14;20)	1 (1.8%)
del (1p)	4 (6.8%)
gain (1q)	23 (39%)
Del (17p)	1 (1.7%)
No genetic profiling result available	15
Number of HRCA	
0	31 (56%)
1	17 (31%)
2+	7 (13%)
Imaging characteristics	
Imaging phenotype at baseline	
Focal & diffuse disease present	46 (65.7%)
Focal disease only	11 (15.7%)
Diffuse disease only	13 (18.6%)
Line and type of treatment	
Newly diagnosed myeloma (NDMM)	54 (77%)
Induction therapy	
VTD	44 (81% of NDMM)
VRD	2 (3.7%)
CTD	3 (5.6%)
CVD	2 (3.7%)
DVD	1 (1.9%)
KCRD	1 (1.9%)
VD	1 (1.9%)
First relapse	16 (23%)
Reinduction therapy	
ITD	7 (44% of those at first relapse)
VTD	2 (13%)
CVD	2 (13%)
CRD	1 (6.3%)
KCD	1 (6.3%)
KD	1 (6.3%)
RD	1 (6.3%)
VD	1 (6.3%)
Time from diagnosis to first relapse (months; median (IQR))	53 (25, 57)
Received post-ASCT maintenance therapy	19 (28%)
Post-ASCT maintenance therapy	
Lenalidomide	8 (42%)
Ixazomib	5 (26%)
IRD	2 (11%)
V	1 (5.3%)
DRD	1 (5.3%)
PomD	1 (5.3%)
VTD	1 (5.3%)
No maintenance therapy/watch and wait	51 (73%)

^1^*n* (%); where no data available for some patients, (%) refers to the denominator with available information; median (Q1, Q3).

### Serological response, bone marrow minimal residual disease assessment and imaging response

Induction therapies received by patients pre-ASCT are listed in Table 1. For both NDMM and patients at first relapse, induction triplet regimens with proteasome inhibitor (PI), immunomodulatory drugs (IMiDs) and dexamethasone combinations were commonest; post-ASCT, 28% received maintenance therapy (Table 1).

Of the 70 included patients, 35 (51%) reached a complete response (CR) or stringent CR (sCR) as per IMWG response criteria, and 20 (30%) very good partial response (VGPR) and 13 (19%) a partial response (PR) at day 100 post-ASCT (Table 2).

**Table 2:** Post-autologous stem cell therapy response to treatment.

Characteristic	*N* = 70^1^
IMWG response	
sCR	15 (22%)
CR	20 (29%)
VGPR	21 (30%)
PR	13 (19%)
Unknown^1^	
MRD assessment	
Negative	33 (60%)
Positive	22 (40%)
Unknown	15
MY-RADS assessment	
RAC1	47 (67%)
RAC2 or higher	23 (33%)

^1^n (%).

Bone marrow minimal residual disease (MRD) was assessed, irrespective of serological response, at day 100 post-ASCT by flow cytometry, with a sensitivity of 10^−4^. Fifty-five of the 70 patients had an evaluable bone marrow MRD result. Using patients with an interpretable MRD result as the denominator, since the aim of this study was exploratory, not to test efficacy of the chemotherapy regimen, 60% of patients had undetectable, and 40% detectable MRD at day 100 post-ASCT (Table 2).

On WB-MRI at day 100 post-ASCT, no imaging disease activity as per MY-RADS (RAD1) was reported in 47 (67%) patients, whereas 23 (33%) patients showed disease activity (RAD2 or higher).

Although there was partial overlap, there was no significant correlation between imaging response by MY-RADS and serological response as per IMWG, or bone marrow MRD by flow cytometry (Table 3).

**Table 3:** Inter-relationship of imaging response, MRD and serological response assessments.

A				
	Bone marrow MRD		Total	*p* value^*1*^
	Negative	Positive		
Imaging response				0.3
RAC1	24 (44%)	13 (24%)	37 (67%)	
RAC2+	9 (16%)	9 (16%)	18 (33%)	
Total	33 (60%)	22 (40%)	55 (100%)	

B				
	IMWG response		Total	*p* value^*1*^
	sCR/CR	<CR		
Imaging response				0.5
RAC1	25 (36%)	22 (32%)	47 (68%)	
RAC2+	10 (14%)	12 (17%)	22 (32%)	
Total	35 (51%)	34 (49%)	69 (100%)	

C			
Characteristic	HR^*1*^	95% CI^*1*^	*p* value
Imaging response			
RAC1	—	—	
RAC2+	2.08	1.01, 4.31	0.048
IMWG response			
CR	—	—	
<CR	2.00	0.98, 4.08	0.057
2 + HRCA	1.43	0.89, 2.28	0.14

^*1*^Pearson’s Chi-squared test.

*HR* Hazard ratio, *CI* confidence interval.

Cross tables for A bone marrow MRD and imaging response as per MY-RADS (*n* = 55 patients with both variables) B IMWG serological and imaging response (*n* = 70). Multivariable Cox-regression analysis C of imaging response assessment in a model including serological response and HRCA (*n* = 65).

### Association of imaging response with outcome

**Fig. 2:**
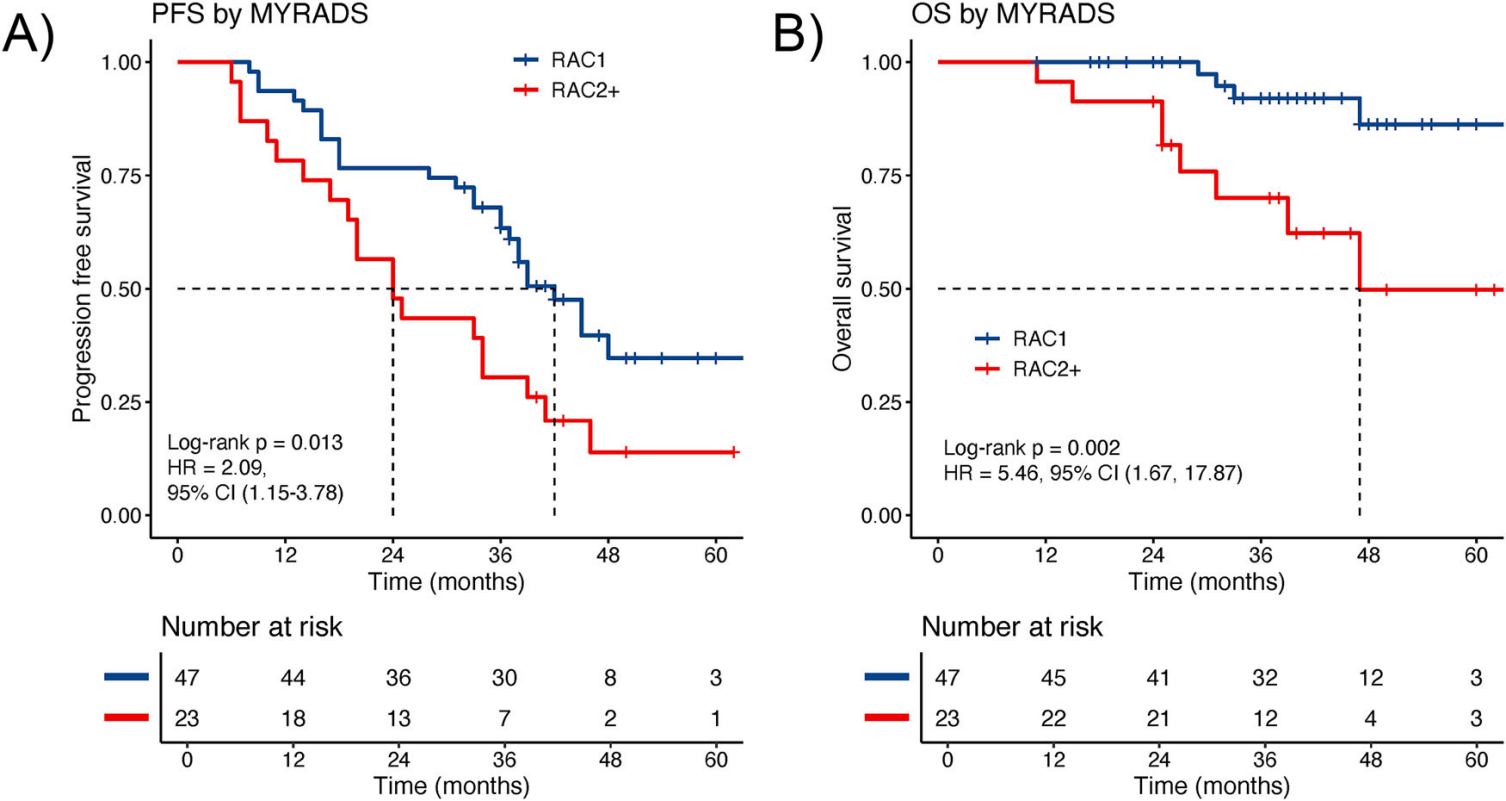
Prognostic association of imaging response as per MY-RADS. **A** Kaplan–Meier curve contrasting the groups with no detectable disease (RAD1) vs patients with detectable disease (RAC2+) on WB-MRI as per MY-RADS criteria at day 100 post-ASCT. **A** for PFS **B** for OS.

We found imaging response by MY-RADS to be significantly associated with patient outcome. Patients with RAC2 or higher had significantly shorter PFS than those with RAC1, with median PFS 24 months (95% confidence interval (CI): 16.6–33.9) vs. 42 mo (95% CI: 37—not evaluable (NE)), log-rank *p* = 0.013, and a HR of 2.09 (95% CI: 1.15–3.78) (Fig. 2). OS was also significantly shorter for RAC2 or higher, with median OS 47 mo (95% CI: 30.9–NE) vs. NE (95% CI: NE–NE), *p* = 0.002, HR = 5.45 (95% CI: 1.67–17.87). We performed sensitivity analyses to investigate potential differences in prognostic association for NDMM and patients at relapse. Analysing the NDMM (*n* = 54) separately, imaging response by MY-RADS was significantly associated with PFS and OS in the NDMM patient group, consistent with the main study group results (Supplementary Fig. 1). The relapse group was too small to perform a separate group-wise comparison. MY-RADS imaging response also remained significantly associated with PFS and OS in a Cox multivariable regression analysis that included induction therapy as a model variable (Supplementary Tables 1 and 2).

Serological response was associated with outcome, with patients with less than CR showing inferior PFS to patients in CR or stringent CR, *p* = 0.028, HR = 1.95 (95% CI: 1.06–3.58), but there was no association with shorter OS (Supplementary Fig. 2). Bone marrow MRD status was also associated with shorter PFS for detectable vs undetectable MRD, *p* = 0.0016, HR = 2.94 (95% CI: 1.47–5.89), but no significant difference in OS (Supplementary Fig. 3). We also investigated association of high-risk genetics with outcome and patients with 2 + HRCA had significantly shorter PFS (*p* = 0.024, HR = 1.63 (95% CI: 1.05–2.54) and OS (*p* = 0.018, HR = 2.10 (95% CI: 1.07–4.15) (Supplementary Fig. 4).

When analysing imaging response by WB-MRI in a multivariable Cox-regression model with serological response and high-risk genetics, response by WB-MRI retained its association with PFS (Table 3). Multivariable analysis, including bone marrow MRD was limited, as 15 patients had to be excluded due to missing MRD results. Nevertheless, in this model, there was a trend for imaging response retaining its association with PFS with HR = 1.90 (95% CI: 0.95–3.82), Wald-*p* = 0.07, albeit not meeting pre-defined criteria of significance *(*Supplementary Table 3; Supplementary Figs. 5 and 6). In a sensitivity multivariable Cox-regression analysis of the NDMM patient sub-group (*n* = 54; of which *n* = 43 with MRD data), both imaging response by MY-RADS and MRD were significantly associated with PFS (Supplementary Table 4).

**Fig. 3:**
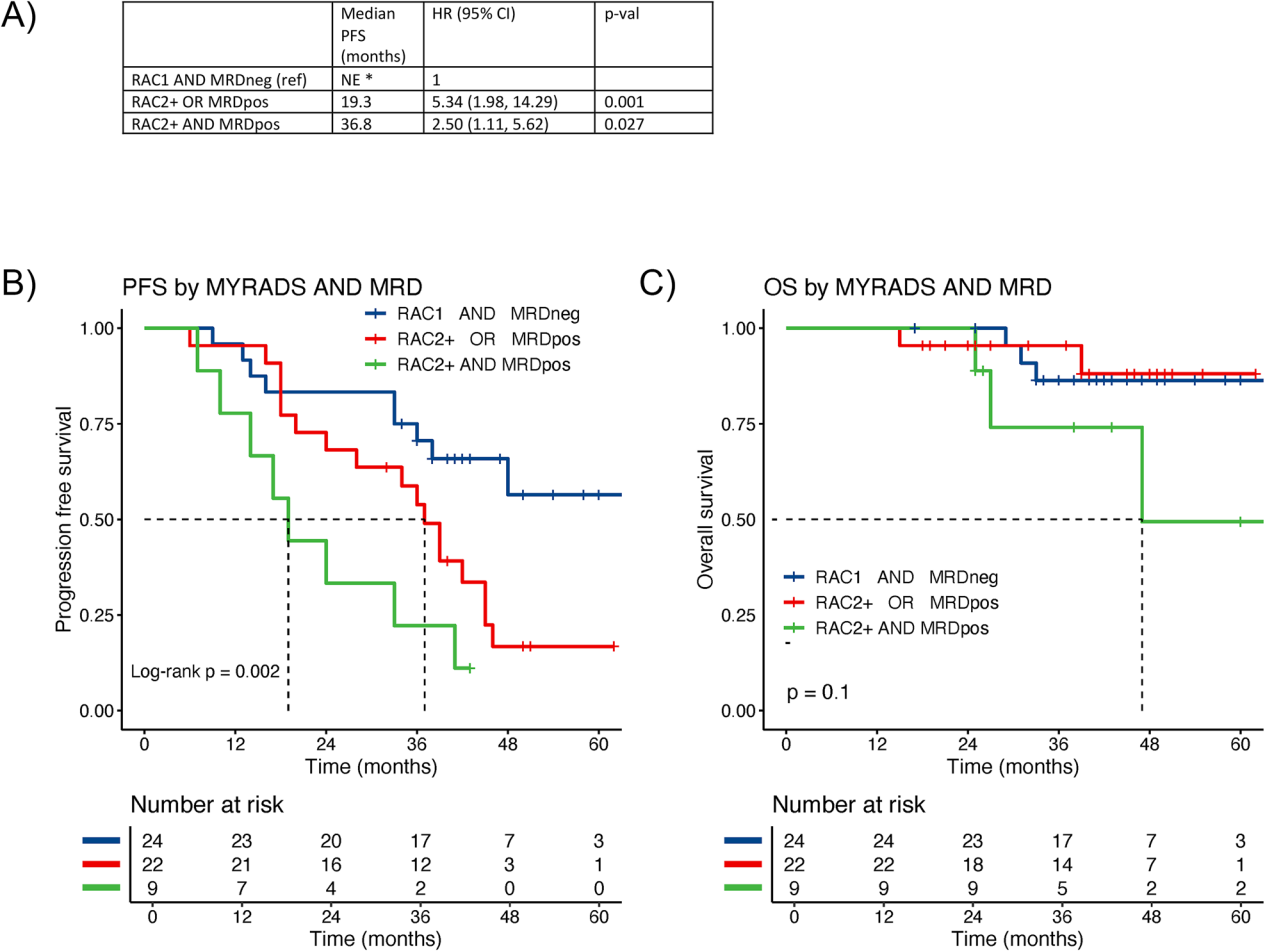
Prognostic association of combined bone marrow MRD and imaging response assessment. Kaplan–Meier curves contrasting participants with no detectable disease on WB-MRI and bone marrow MRD (RAC1 AND MRD neg) at day 100 post-ASCT, those with detectable disease by either imaging or MRD (RAC2 + OR MRD pos) and those with detectable disease by both methods (RAC2 + AND MRD pos). Summary **A**, Kaplan-Meier curves for PFS **B**, and Kaplan Meier curves for OS **C**.

Combined WB-MRI imaging and bone marrow MRD data were used to create three response strata: patients with RAC1 AND undetectable MRD (RAC1 MRD−), RAC2 + OR detectable MRD (RAC2 + OR MRD+) and RAC2 + AND detectable MRD (RAC2+ AND MRD+). PFS was poorest for the group with RAC2 + MRD+, with median 19.3 mo (95% CI: 19 – NE), HR = 5.34 (1.98, 14.29), Wald-*p* = 0.001, compared to RAC1 MRD- with median PFS that was NE, with RAC2 + OR MRD+ showing an intermediate outcome with median PFS 36.8 mo (95% CI: 28–46), HR = 2.50 (95% CI: 1.11–5.62), Wald-*p* = 0.027 (Fig. 3). Although follow-up was relatively short, limiting OS analyses, the group with RAC2 + MRD+ showed nominally the shortest median OS of 47 mo (95% CI; 27–NE), whilst median OS was NE for the other two groups (Fig. 3). A similar result was seen when combining WB-MRI response with serological response (Supplementary Fig. 7).

## Discussion

The benefits of synergistic evaluation of residual disease through bone marrow MRD laboratory testing and imaging are now well established because the irregular distribution of plasma cells in the marrow space results in discordance between the techniques [4, 17]. Compelling evidence from prospective FDG PET/CT studies, therefore, has informed the IMWG recommendation for FDG PET/CT assessment of residual disease [1, 4, 18, 19]. However, the superior sensitivity of contemporary WB-MRI protocols over FDG PET/CT is now established in the diagnostic setting [7–9, 13, 20]. Furthermore, the international consensus Myeloma Response Assessment and Diagnosis System (MY-RADS) imaging recommendations now provide freely downloadable scanner protocols for all vendors, and standardisation for acquisition, interpretation and reporting of WB-MRI in myeloma, allowing unified response assessment. In addition, quality-controlled MY-RADS acquisition has been validated in a prospective multivendor multicentre study [21, 22]. Nevertheless, prospective evidence on the prognostic relevance of residual disease on WB-MRI has been lacking so far. Our results demonstrate, to our knowledge, for the first time in a prospective trial specifically set up for this purpose, an association of residual disease activity by WB-MRI performed with functional imaging techniques as per MY-RADS criteria with shorter PFS and shorter OS.

These findings are essential evidence for its use in standard care and clinical trials and are in keeping with landmark retrospective analyses such as that by Rasche et al., which explored FDG PET/CT and MRI [23, 24]. In this study, residual focal lesions were detected in 24% of first-line patients and were associated with short progression-free survival, with MRI detecting more focal lesions than FDG PET/CT in some patients. Furthermore, the combination of MRD and imaging improved outcome prediction. Although residual focal lesions were rare in MRD-negative first-line patients (12%), this was more common following salvage therapy (50%).

The introduction of functional imaging techniques into WB-MRI protocols has not only increased sensitivity and specificity of disease detection, but it has also enabled assessments of response independent of changes in lesion size [25–27]. Anatomical sequences remain relevant for anatomy and assessment of mechanical complications of myeloma, but diffusion-weighted MRI is not only highly sensitive but also allows generation of quantitative apparent diffusion coefficient maps (ADC), which are inversely proportional to cellularity, i.e., as a lesion responds to treatment, the ADC rises. ADC maps also contribute to specificity as previously treated sites of disease can persist on anatomical sequences, but the ADC will distinguish treated from active/cellular sites. Furthermore, benign bone lesions such as haemangiomas have different characteristics from disease [28]. Dixon MRI also supports disease detection and generates quantitative fat fraction maps, allowing for changes in marrow fat to contribute to response assessments as normal marrow fat becomes replaced by disease, but can repopulate when disease responds [25]. The data presented here provide the necessary clinical context for these technical parameters that show high sensitivity and good repeatability and reproducibility [29–31]. These technical features of WB-MRI add to the principal advantage of sparing patients' exposure to ionising radiation, which gains relevance in the context of increasing long-term survival of patients.

MY-RADS also included a scoring system for response, the RAC criteria. In 2021, Belotti et al. published the first retrospective validation of the RAC criteria in 64 newly diagnosed myeloma patients [32]. Superior post-autograft PFS and OS were observed in patients with complete imaging response (RAC1), and combining MRD and imaging improved the prediction of outcome. A three-year post-autograft OS of 92% was reported for patients with RAC1 versus 69% for those with RAC ≥ 2 (*p* = 0.047). Importantly, radiologists did not report difficulties in applying MY-RADS guidelines [32].

Strengths of our analyses include the prospective design, real-world treatment and diagnostic setting, including diagnostic-grade bone marrow MRD and genetic characterisation. Limitations include the single-centre design and, consequently, the use of one MRI vendor. However, we have recently developed quality control algorithms for data control across vendors in multicentre studies, ensuring future transferability of results [21]. Furthermore, the study was performed prior to introduction of anti-CD38 therapy; however, most patients received triplet PI/IMiD induction therapy, and the prognostic association of assessments such as bone marrow MRD and imaging response by FDG PET/CT has generally been demonstrated to be therapy-agnostic, including consistency across anti-CD38 and non-anti-CD38 containing regimens [4, 16, 17, 33]. Although MRD assessment was performed at 10^−4^ sensitivity, which would preferentially be superseded by 10^−5^ assessment in contemporary individual patient care, its prognostic association, even at lower levels, is still consistent at lower sensitivity levels [33]. Seventy of 105 included patients were evaluable in our study, based on pre-defined criteria. Reasons for non-evaluability were due to a variety of factors, mostly induction treatment-related, such as early relapse, loss to follow-up, change in performance status, failure of harvesting and other factors typical for a real-world treatment cohort. In only 5 patients was the reason that no post-ASCT response WB-MRI was performed, suggesting a generally high deliverability of imaging response assessment by WB-MRI. We also have, since 2022 (after trial closure), implemented WB-MRI imaging response assessment post-ASCT as part of the standard of care first-line management for all patients treated at our centre, with virtually all patients undergoing ASCT also being able to undergo imaging response assessment in our experience.

In summary, our results propose WB-MRI as a new standard for myeloma tumour imaging and support its wider implementation in standard care and clinical research for the benefit of patients.

## Supplementary information

Supplementary material

## Data Availability

The datasets generated during and/or analysed during the current study are available from the corresponding author on reasonable request.
