# Supplementary material for: Whole body MRI by MY-RADS for imaging response assessment in multiple myeloma
Source: Blood Cancer J. 2025 Jul 17;15(1):122. doi: 10.1038/s41408-025-01327-4 (PMC12271311; doi:10.1038/s41408-025-01327-4)

**SUPPLEMENTARY MATERIAL**

**Legends**

**Supplementary Table 1. Multivariable Cox model for Progression-Free Survival – including (re-)induction therapy (n=70 patients)**

**Supplementary Table 2. Multivariable Cox model for Overall Survival – including (re-)induction therapy (n=70 patients)**

**Supplementary Table 3. Multivariable Cox model for Progression-Free Survival – including bone marrow MRD (n=55 patients)**

**Supplementary Table 4. Multivariable Cox model for Progression-Free Survival – including bone marrow MRD in NDMM patients only (n=43 patients)**

**Supplementary Figure 1. Patient outcome in relation to imaging response at day 100 post-ASCT in the sub-group of newly diagnosed patients.** A) PFS B) OS.

**Supplementary Figure 2. Patient outcome in relation to IMWG response at day 100 post-ASCT.** A) PFS B) OS.

**Supplementary Figure 3. Patient outcome in relation to bone marrow MRD at day 100 post-ASCT.** A) PFS B) OS.

**Supplementary Figure 4. Patient outcome in relation to high-risk genetics, specifically presence of 2 or more HRCA.** A) PFS B) OS.

**Supplementary Figure 5. Persistent active disease post autologous stem cell transplantation in an MRD positive patient.** Whole body MRI at baseline (A-E) and 3 months post autologous stem cell transplantation (F-J) at which time testing was positive for MRD. At baseline the b900 MIP (A) demonstrated multiple focal lesions including focal lesions in the left pelvis highlighted on the b900 (B,D) diffusion weighted images and relative ADC maps (D,E) which showed low ADC in keeping with focal active sites of disease. Three months (D100) post autologous stem cell transplantation the lesion in the anterior left pelvis (G,H) maintained a low ADC (H) suggesting persistent cellular active disease. The lesion in the posterior left acetabulum (I,J) returns a very high ADC (J) equivalent to fluid in the bladder (*) in keeping with treated acellular lesion.

**Supplementary Figure 6. Persistent active disease post autologous stem cell transplantation in an MRD negative patient.** Whole body MRI at baseline (A-C) and 3 months (D100) post autologous stem cell transplantation (D-F) at which time testing was negative for MRD. At baseline the b900 MIP (A) demonstrated multiple focal lesions including focal lesions in the thoracic spine (arrows). Axial b900 diffusion weighted MRI highlights the focal lesion in the proximal thoracic spine (B) which has a low ADC (C) in keeping with focal active disease. Three months post autologous stem cell transplantation the more inferior thoracic spine lesion has disappeared (D), but the superior thoracic spine lesion persists on the b900 mip and axial b900 diffusion weighted MRI (D-E) and retains a low ADC (F) in keeping with persistent focal active disease.

**Supplementary Figure 7. Group stratification based on a combination of WB-MRI imaging response and IMWG response at day 100 post-ASCT.** A) PFS B) OS.

**Supplementary Table 1. Multivariable Cox model for Progression-Free Survival – including (re-)induction therapy (n=70 patients)**

| **Characteristic** | **HR***^1^* | **95% CI***^1^* | **p-value** |
| --- | --- | --- | --- |
| Imaging response |  |  |  |
| RAC1 | — | — |  |
| RAC2+ | 2.11 | 1.16, 3.83 | 0.014 |
| Induction therapy^2^ |  |  |  |
| IMID | — | — |  |
| PI | 0.87 | 0.23, 3.27 | 0.8 |
| PI & IMD or anti-CD38-PI | 0.79 | 0.28, 2.24 | 0.7 |
| *^1^* HR = Hazard Ratio, CI = Confidence Interval  ^2^ IMID=immunomodulatory drug based combination (thalidomide, lenalidomide), PI=proteasome inhibitor based combination | | | |

**Supplementary Table 2. Multivariable Cox model for Overall Survival – including (re-)induction therapy (n=70 patients)**

| **Characteristic** | **HR***^1^* | **95% CI***^1^* | **p-value** |
| --- | --- | --- | --- |
| Imaging response |  |  |  |
| RAC1 | — | — |  |
| RAC2+ | 5.82 | 1.77, 19.2 | 0.004 |
| Induction therapy^2^ |  |  |  |
| IMID | — | — |  |
| PI | 1.56 | 0.14, 17.3 | 0.7 |
| PI & IMD or anti-CD38-PI | 0.78 | 0.10, 6.24 | 0.8 |
| *^1^* HR = Hazard Ratio, CI = Confidence Interval  ^2^ IMID=immunomodulatory drug based combination (thalidomide, lenalidomide), PI=proteasome inhibitor based combination | | | |

**Supplementary Table 3. Multivariable Cox model for Progression-Free Survival – including bone marrow MRD (n=55 patients)**

| **Characteristic** | **HR***^1^* | **95% CI***^1^* | **p-value** |
| --- | --- | --- | --- |
| Imaging response |  |  |  |
| RAC1 | — | — |  |
| RAC2+ | 1.90 | 0.95, 3.82 | 0.070 |
| IMWG response |  |  |  |
| CR | — | — |  |
| <CR | 0.81 | 0.35, 1.91 | 0.6 |
| Bone marrow MRD |  |  |  |
| Negative | — | — |  |
| Positive | 3.29 | 1.40, 7.74 | 0.006 |
| *^1^* HR = Hazard Ratio, CI = Confidence Interval | | | |

**Supplementary Table 4. Multivariable Cox model for Progression-Free Survival – including bone marrow MRD in NDMM patients only (n=43 patients)**

| **Characteristic** | **HR*^1^*** | **95% CI*^1^*** | **p-value** |
| --- | --- | --- | --- |
| Imaging response |  |  |  |
| RAC1 | — | — |  |
| RAC2+ | 2.47 | 1.13, 5.43 | 0.024 |
| Bone marrow MRD |  |  |  |
| Negative | — | — |  |
| Positive | 3.00 | 1.35, 6.67 | 0.007 |
| *^1^* HR = Hazard Ratio, CI = Confidence Interval | | | |

**Supplementary Figure 1. Patient outcome in relation to imaging response at day 100 post-ASCT in the sub-group of newly diagnosed patients.** A) PFS B) OS.

A)


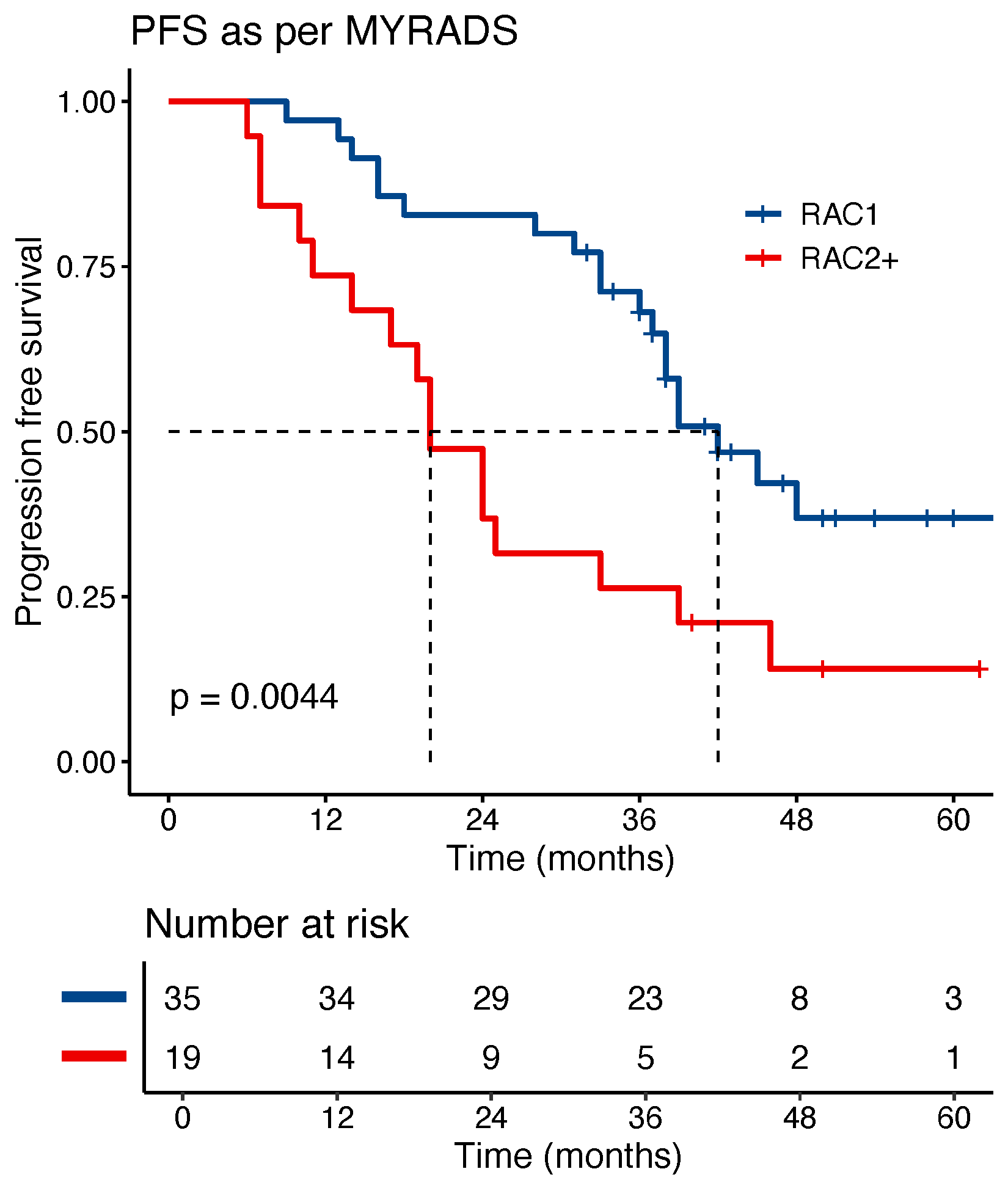


B)


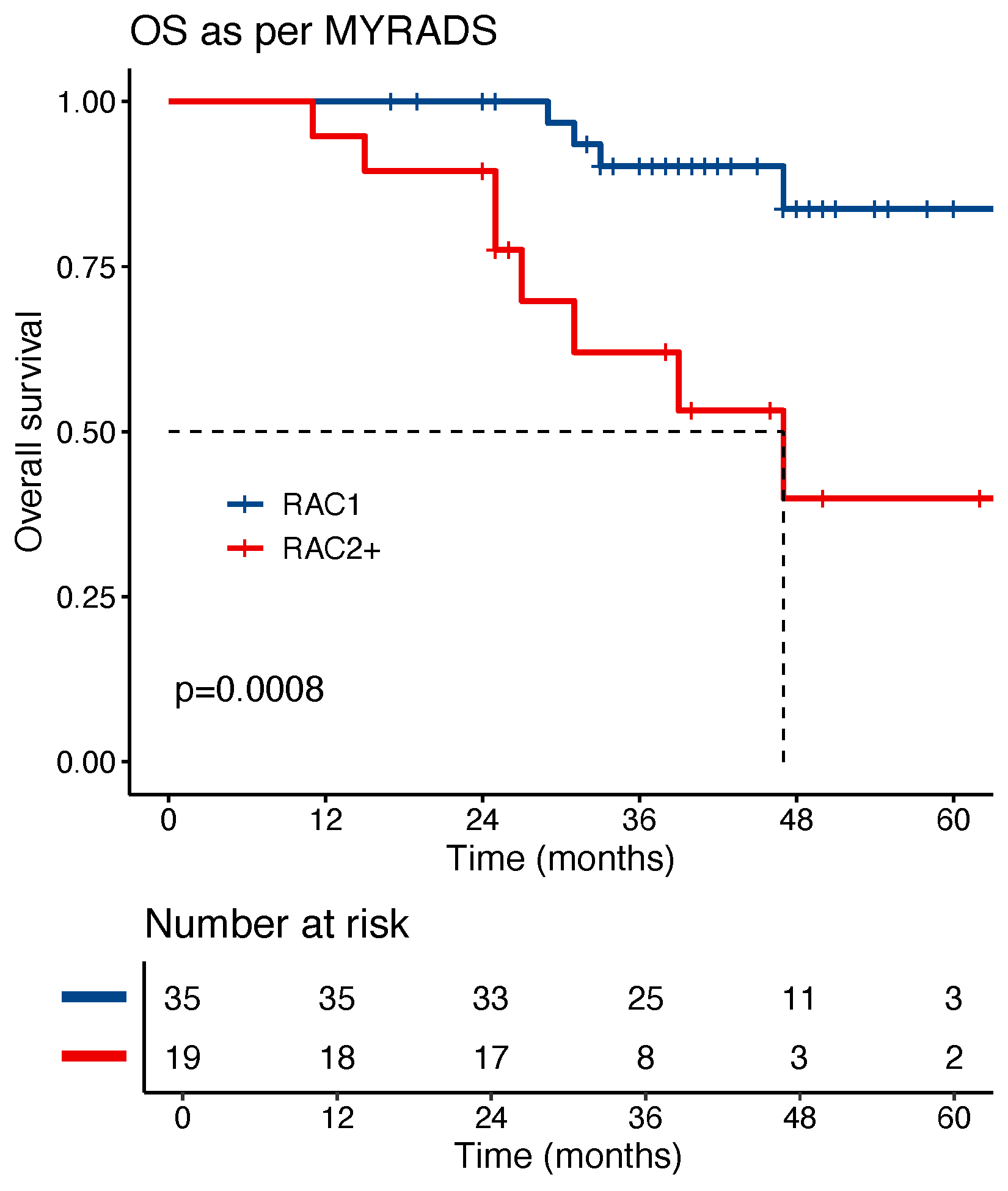


**Supplementary Figure 2. Patient outcome in relation to IMWG response at day 100 post-ASCT.** A) PFS B) OS.

A)


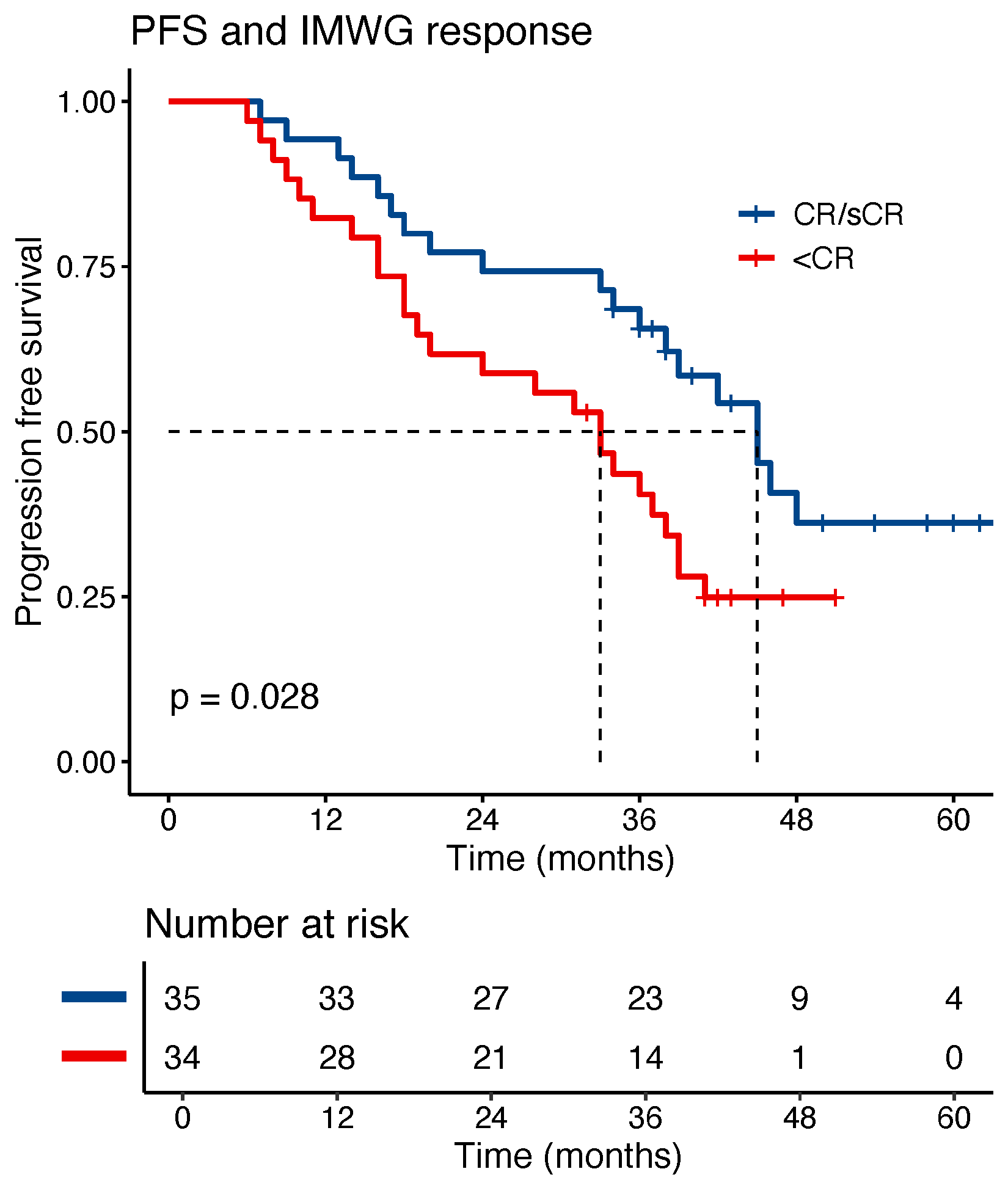


B)


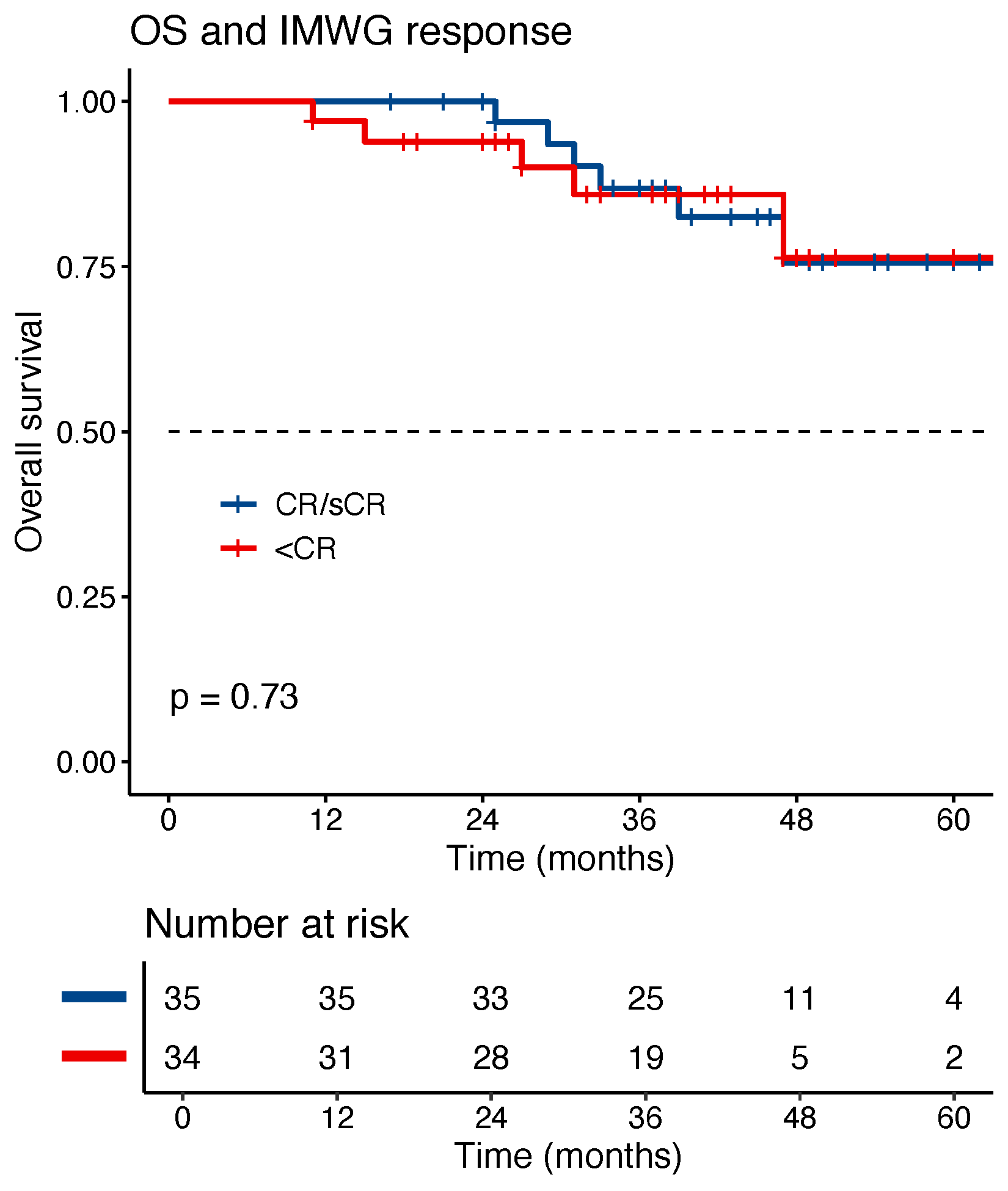


**Supplementary Figure 3. Patient outcome in relation to bone marrow MRD at day 100 post-ASCT.** A) PFS B) OS.

A)


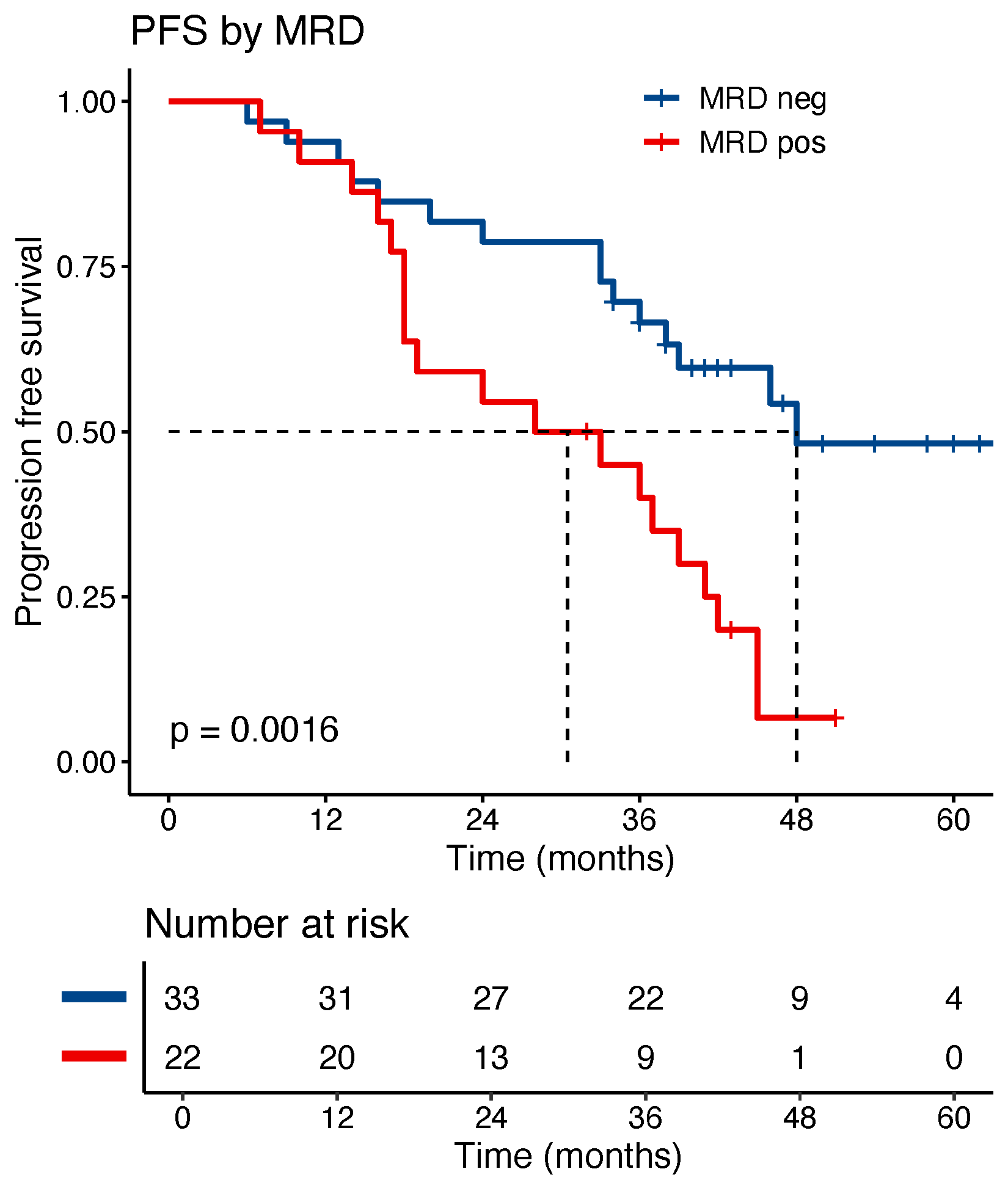


B)


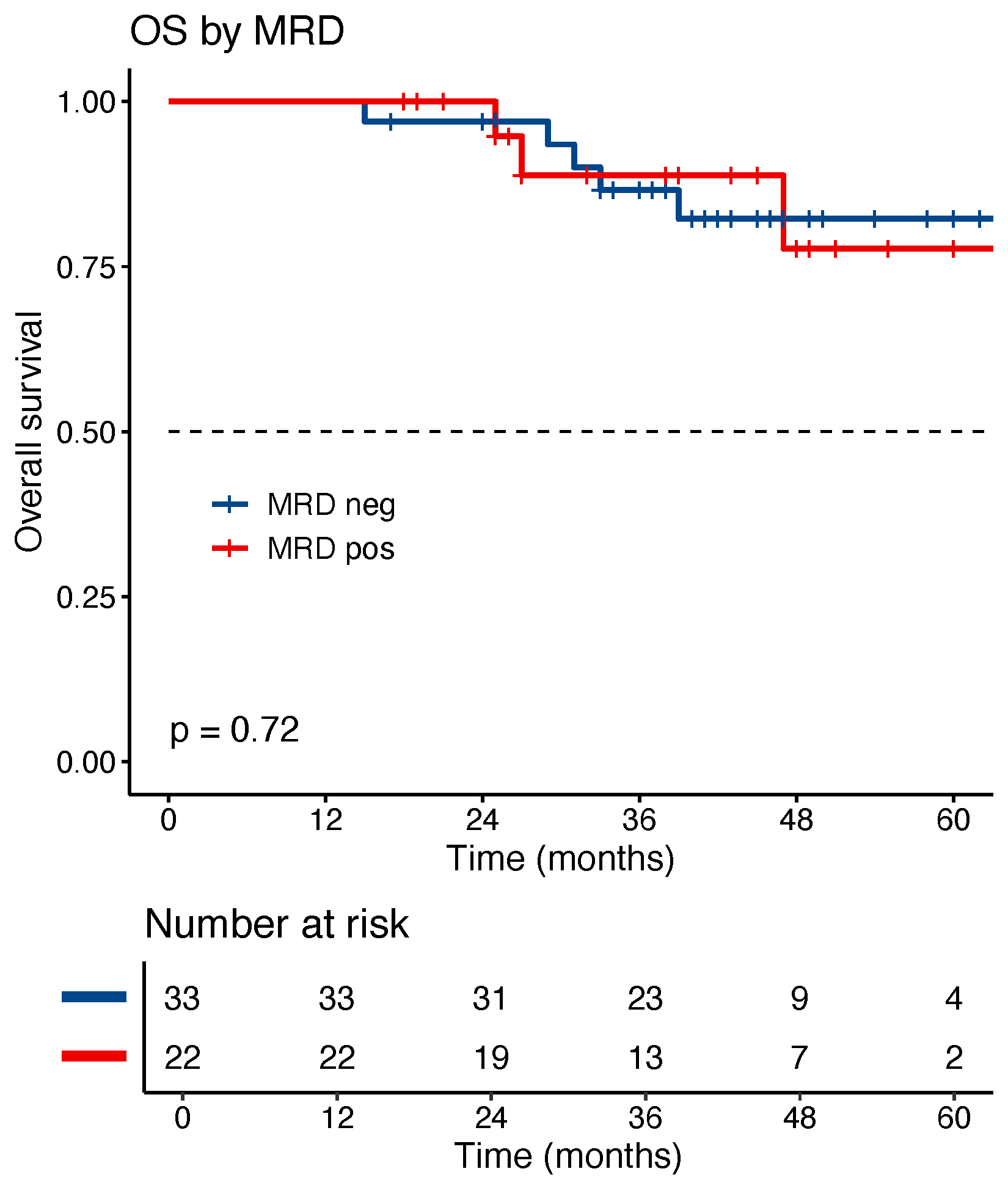


**Supplementary Figure 4. Patient outcome in relation to high-risk genetics, specifically presence of 2 or more HRCA.** A) PFS B) OS.

A)


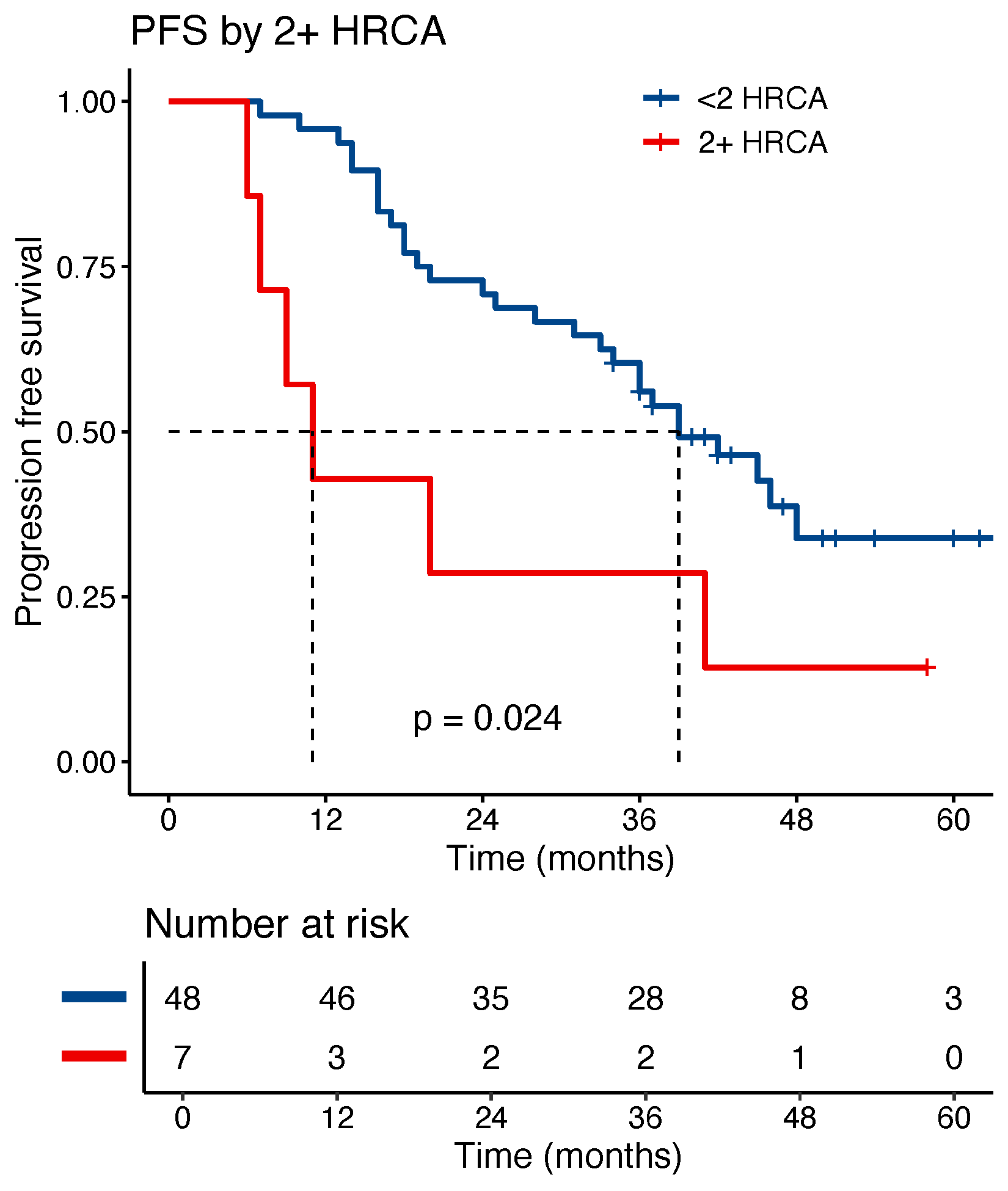


B)


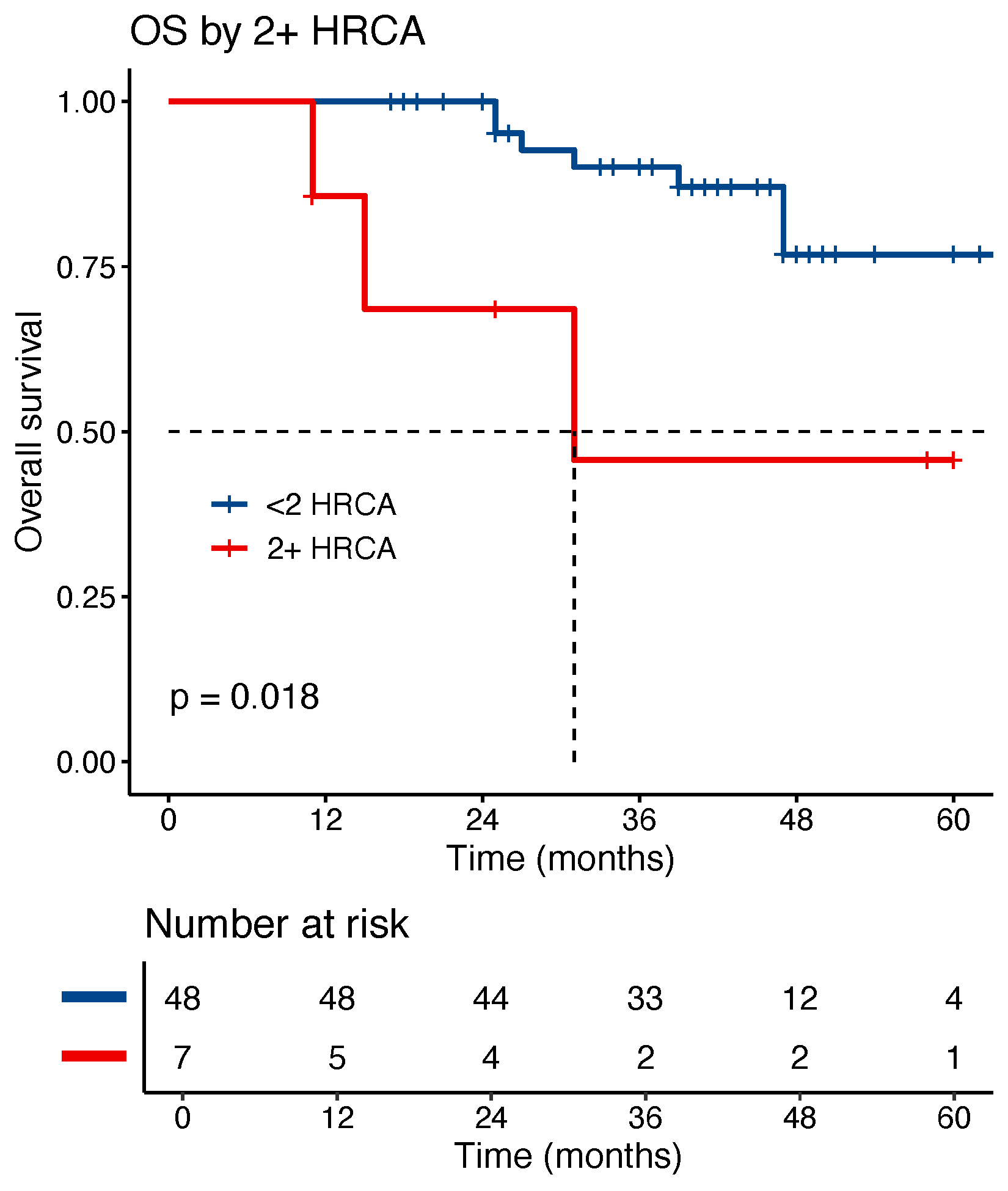


**Supplementary Figure 5. Persistent active disease post autologous stem cell transplantation in an MRD positive patient.** Whole body MRI at baseline (A-E) and 3 months post autologous stem cell transplantation (F-J) at which time testing was positive for MRD. At baseline the b900 MIP (A) demonstrated multiple focal lesions including focal lesions in the left pelvis highlighted on the b900 (B,D) diffusion weighted images and relative ADC maps (D,E) which showed low ADC in keeping with focal active sites of disease. Three months (D100) post autologous stem cell transplantation the lesion in the anterior left pelvis (G,H) maintained a low ADC (H) suggesting persistent cellular active disease. The lesion in the posterior left acetabulum (I,J) returns a very high ADC (J) equivalent to fluid in the bladder (*) in keeping with treated acellular lesion.


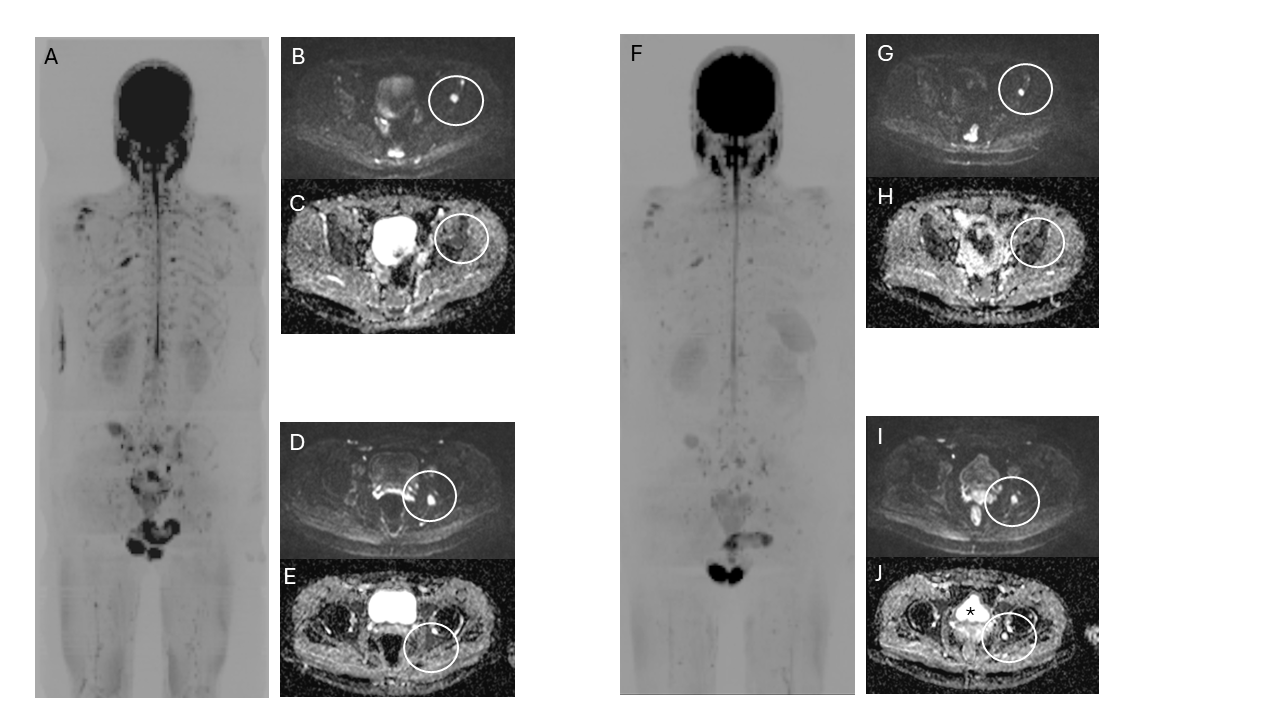


**Supplementary Figure 6. Persistent active disease post autologous stem cell transplantation in an MRD negative patient.** Whole body MRI at baseline (A-C) and 3 months (D100) post autologous stem cell transplantation (D-F) at which time testing was negative for MRD. At baseline the b900 MIP (A) demonstrated multiple focal lesions including focal lesions in the thoracic spine (arrows). Axial b900 diffusion weighted MRI highlights the focal lesion in the proximal thoracic spine (B) which has a low ADC (C) in keeping with focal active disease. Three months post autologous stem cell transplantation the more inferior thoracic spine lesion has disappeared (D), but the superior thoracic spine lesion persists on the b900 mip and axial b900 diffusion weighted MRI (D-E) and retains a low ADC (F) in keeping with persistent focal active disease.

B)

**
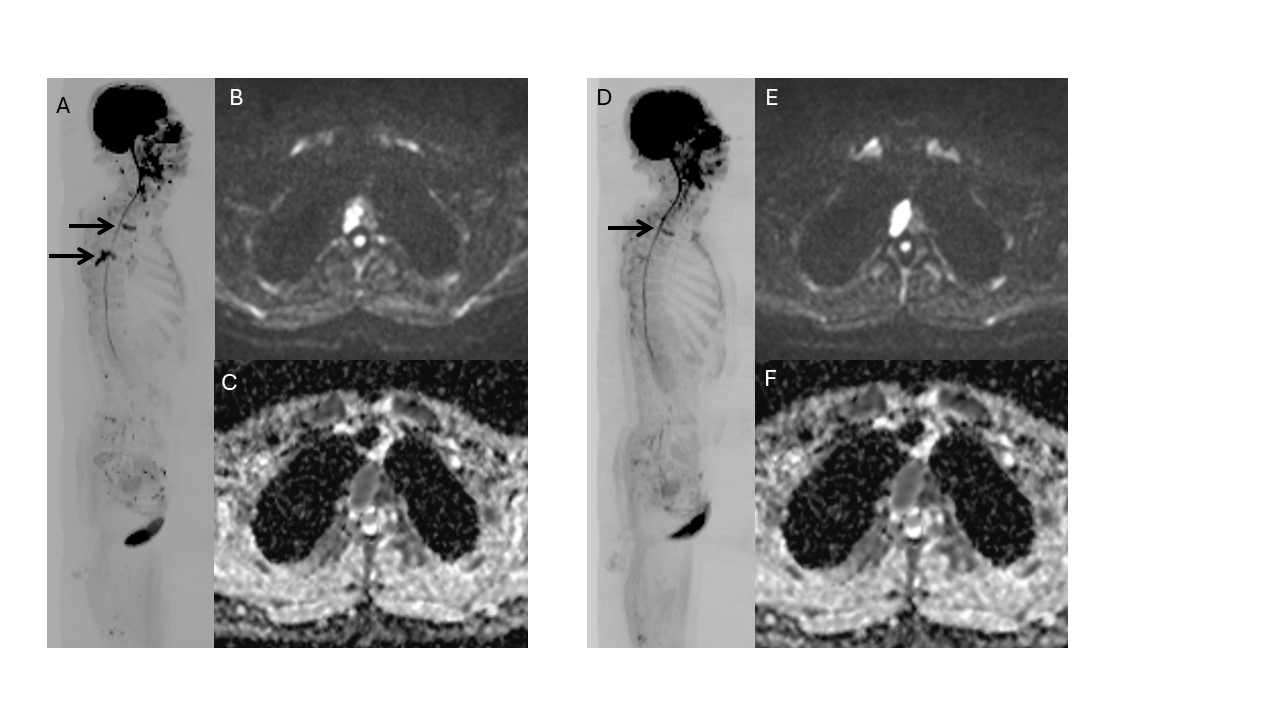
**

**Supplementary Figure 7. Group stratification based on a combination of WB-MRI imaging response and IMWG response at day 100 post-ASCT.** A) PFS B) OS.

A)


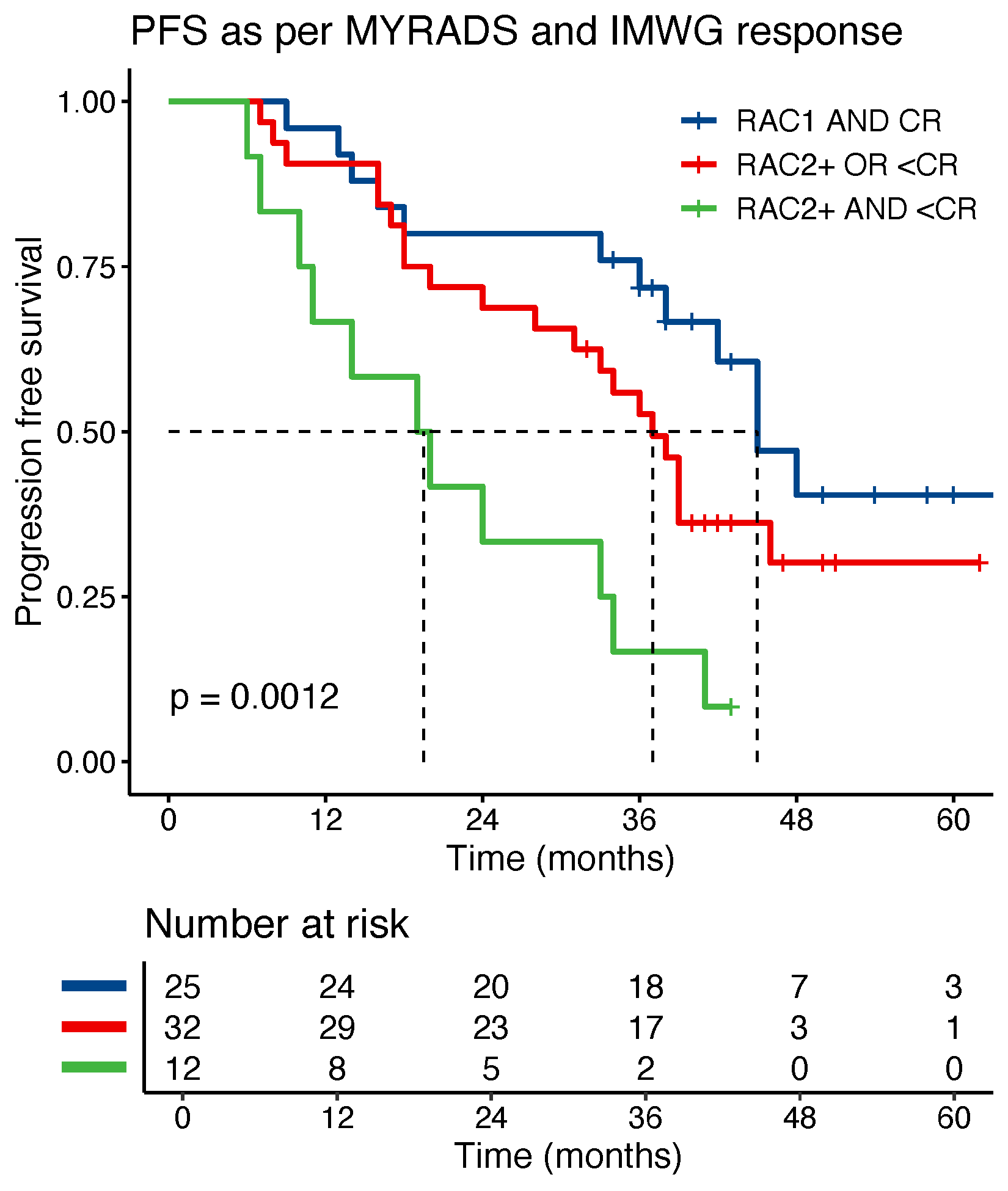


B)


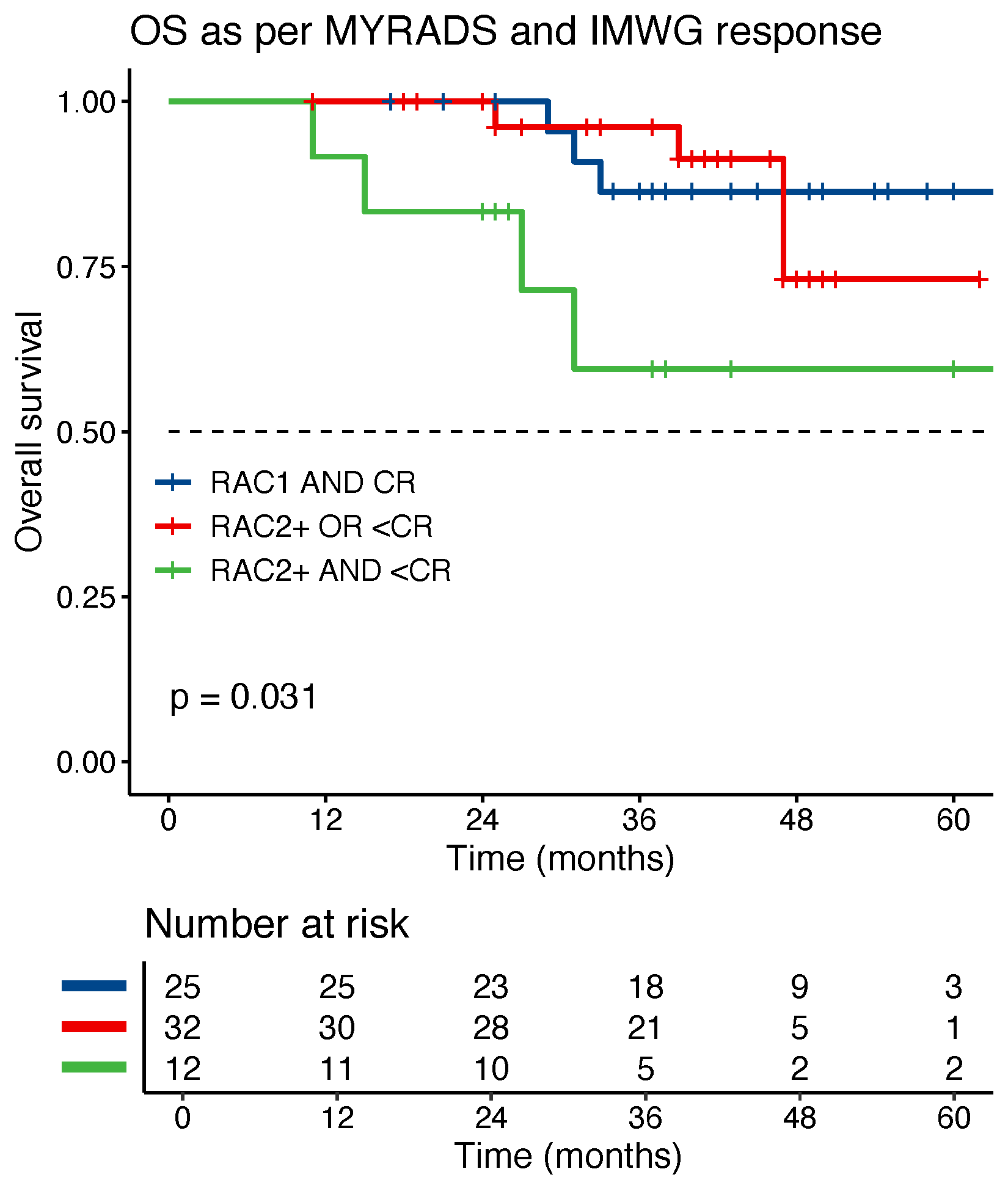

Supplement: Supplementary file 1 — Supplementary material [file 41408_2025_1327_MOESM1_ESM.docx]
